# A Constitutive Material Model Applied to Microforming Processes

**DOI:** 10.3390/ma13225143

**Published:** 2020-11-15

**Authors:** Zbigniew Zimniak

**Affiliations:** Department of Metal Forming, Welding and Metrology, Wrocław University of Science and Technology, ul. Łukasiewicza 5, 50-371 Wrocław, Poland; zbigniew.zimniak@pwr.edu.pl; Tel.: +48-713-202-162

**Keywords:** constitutive model, microforming, size effect, FEM

## Abstract

The plastic treatment of products reduced to sizes corresponding to the microscale poses difficulties, due to the occurrence of the so-called size effect, which is responsible for the different behavior of the material during the realization of microforming. In this study, a constitutive equation was elaborated taking into account two types of size effects, with the use of a surface model as well as a composite material model. The influence of the size effect referring to both the material grain size and the geometric scaling of the sample size on the material’s flow stresses was considered. The surface model took into account the different grain shapes present in actual polycrystalline materials. After the application of the presented model for titanium Grade 2, a good agreement of the experimental results with the FEM simulation results was obtained. Thus, the proper FEM modeling of microforming processes should be conducted with the use of a material model, taking into account the occurring size effects.

## 1. Introduction

One of the modern plastic treatment technologies is microforming, which makes it possible to produce metal components with dimensions below 1 mm [1,2,3,4,5,6,7,8]. At this scale, there are various factors affecting the deformation behavior, process performance, and quality of microformed parts. Microstructures of a material become increasingly important as the size of the material is reduced to the microscale. When sheet metal is only a few grains thick, its mechanical properties may differ from those of the bulk metal with a regular scale. An important issue in the examination of microforming processes is the so-called size effect. Size effects can be described as deviations from the expected result, which take place when the size of the examined object changes. They play a significant role in processes involving scaling. Size effects can be helpful in the development of knowledge as well as in the understanding of the phenomena occurring in micro- and nanotechnology. It was observed that a change in the geometrical dimensions of the sample also causes a change in its mechanical properties [8].

Various theories have been developed for the modeling of macroscale plastic deformation during metal forming. The traditional constitutive model is no longer valid for accurate analysis of microforming processes. In order to obtain a relation between the material’s plastic deformation and the size effects, investigations referring to the microforming of copper and aluminum alloys were performed [9]. The results obtained by the authors showed that the yield stresses decrease with an increasing miniaturization, with a constant grain size of the material [10]. Miyazaki et al. [11] investigated the effect of specimen size on the flow stress of a rod specimen of polycrystalline Cu–Al alloy and an affected zone model was proposed to explain why the flow stress decreases with the dimensional reduction of the specimen. Kals et al. [12] and Engel et al. [13] proposed a model called the “surface model”, which is currently applied to analyze the influence of the size effect on yield stresses. The model assumes that the sample consists of two separate parts (the internal part and the surface layer). The percentage share of the surface layer grains increases with an increase in the grain size or with a decrease in the geometrical dimensions of the object [12,13].

The study presented below uses a model which consists of a surface model [13] and a composite model of polycrystalline materials [14]. The composite model [14] assumes that grains are spherical and are composed of grain boundary layers and grain interiors. This model has been proven to be a valid and effective approach to describe the relationship between the flow stress of the polycrystalline aggregate and grain size.

The model takes into account the phenomenon of grain boundary hardening of the external grain surface under the effect of plastic deformations. The yield stresses in the given grain change according to the “mixture law” [11].

The model proposed in the study takes into consideration two size effects: one connected with the grain size and the other related to the geometrical dimensions of the sample. Experimental studies were conducted on cylindrical samples made of pure titanium Grade 2, which were deformed as samples in a uniaxial compression test. Pure titanium is often used for the manufacture of microparts in biomedical devices and implants because of its light weight, biocompatibility, and outstanding corrosion resistance. The obtained experimental results were then analyzed using the proposed model taking into account the two size effects. The model was then applied in FEM calculations. Next, the experimental results were compared with the results of numerical simulations. The simulations were helpful in the development of tools for microforming implants for a total ossicular replacement prosthesis.

## 2. The Grain Size Effect

**Figure 1:**
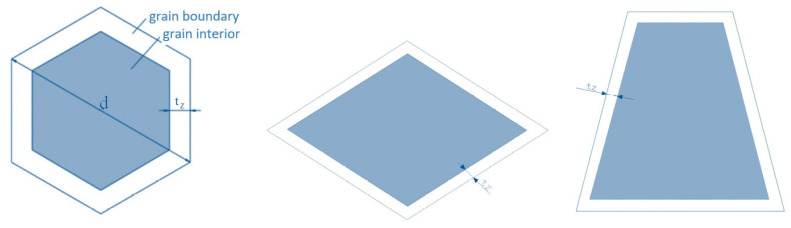
Grain interior and grain boundary models.

For the material taking into account the size effect referring to grain size, the composite material model by Kocks [15] and Meyers and Ashworth [14] was applied. The test showed that, in the given grain, the grain boundary zone exhibits better hardening than its interior zone [16]. Meyers and Ashworth [14] divided the grain into two parts (the grain interior and the grain boundary). This division has been presented in Figure 1 for a few selected grain shapes: a regular hexagon, a rhomb, and a symmetric trapezium, assuming that the grain size is d and the thickness of the grain boundary layer is *t_z_*.

According to the material composite model, the yield stresses are calculated from the equation

(1) $$\sigma_{S}=\sigma_{ZW}f_{ZW}+\sigma_{ZZ}f_{ZZ}$$

where: $\sigma_{S}\;$—the flow stresses of the polycrystalline aggregate (MPa), $f_{ZW},f_{ZZ}\;$—the area fraction of the interior and boundary grains, and $\sigma_{ZW},\sigma_{ZZ}\;$—the flow stresses occurring for the interior and boundary grains (MPa).

The area fractions for the grain model with a regular hexagon shape are as follows:

(2) $$f_{ZW}=\frac{\frac{3\sqrt{3}}{2}{\left(\frac{d}{2}-\frac{2t_{Z}}{\sqrt{3}}\right)}^{2}}{\frac{3\sqrt{3}}{2}{\left(\frac{d}{2}\right)}^{2}}=1-\frac{8\;}{\sqrt{3}}\frac{t_{Z}}{d}+\frac{16}{3}\frac{t_{Z}{}^{2}}{d^{2}}$$

(3) $$\;f_{ZZ}=\frac{\frac{3\sqrt{3}}{2}{\left(\frac{d}{2}\right)}^{2}-\frac{3\sqrt{3}}{2}{\left(\frac{d}{2}-\frac{2t_{Z}}{\sqrt{3}}\right)}^{2}}{\frac{3\sqrt{3}}{2}{\left(\frac{d}{2}\right)}^{2}}=1-f_{ZW}=\frac{8\;}{\sqrt{3}}\frac{t_{Z}}{d}-\frac{16}{3}\frac{t_{Z}{}^{2}}{d^{2}}\;\;\;\;\;\;\;\;\;\;\;\;\;\;\;\;$$

After the substitution of Equations (2) and (3) into Equation (1), the flow stress for the polycrystalline aggregate will equal

(4) $$\sigma_{s}=\sigma_{ZW}+\frac{8}{\sqrt{3}}t_{Z}\left(\sigma_{ZZ}-\sigma_{ZW}\right)d^{-1}-\frac{16}{3}t_{Z}{}^{2}\left(\sigma_{ZZ}-\sigma_{ZW}\right)d^{-2}$$

Taking into account the fact that the interior grain and the boundary grain have different surface fractions in different sections, the mean values for *t_z_* and d were applied:

(5) $$\sigma_{s}=\sigma_{ZW}+\frac{8}{\sqrt{3}}\bar{t_{Z}}\left(\sigma_{ZZ}-\sigma_{ZW}\right){\bar{d}}^{-1}-\frac{16}{3}{\bar{t_{Z}}}^{2}\left(\sigma_{ZZ}-\sigma_{ZW}\right){\bar{d}}^{-2}$$

The dependence between $\bar{d}$ and *d*, as well as between $\bar{t_{z}}$ and *t_z_*, is as follows [14]:

(6) $$\bar{d}=\frac{\pi }{4}d;\;\bar{t_{Z}}=1.57\;t_{Z}\;$$

Therefore,

(7) $$\sigma_{s}=\sigma_{ZW}+\frac{16}{\sqrt{3}}t_{Z}\left(\sigma_{ZZ}-\sigma_{ZW}\right)d^{-1}-\frac{64}{3}t_{Z}{}^{2}\left(\sigma_{ZZ}-\sigma_{ZW}\right)d^{-2}$$

The thickness of the boundary grain layer is connected with the grain size in the following formula [17]:

(8) $$t_{Z}=k\cdot d^{n}(0<n<1)$$

where *k* and *n* are treated as constants for the given material, which can be obtained from the Hall–Petch relation, where, for titanium, *k* = 0.4, and *n* = 0.5 [10].

After the substitution of Equation (9) into Equation (8), the following relation was obtained:

(9) $$\sigma_{s}=\sigma_{ZW}+\frac{16}{\sqrt{3}}0.4\left(\sigma_{ZZ}-\sigma_{ZW}\right)d^{-0.5}-\frac{64}{3}{\left(0.4\right)}^{2}\left(\sigma_{ZZ}-\sigma_{ZW}\right)d^{-1\;}$$

Similar considerations can be performed for a grain with a rhomb and symmetric trapezium shape, which can be isolated in titanium Grade 2. It was assumed that in the case of a rhomb, its shorter diagonal is equal to the diameter of the grain d. It was assumed that the angles in the rhomb equal 65° and 115°, whereas the thickness of the external grain layer *t_z_* was calculated based on trigonometrical equations. The area fractions of the particular parts of the grain equal

(10) $$f_{ZW}=\frac{\frac{1}{2}\left(d-\frac{2\;tz}{\sin57.5{}^{\circ}}\right)\left(\frac{d\pi }{2}-\frac{2tz}{\sin32.5{}^{\circ}}\right)}{\frac{1}{2}d\cdot \frac{d\pi }{2}}$$

(11) $$\;f_{ZZ}=\frac{\frac{d^{2}\pi }{2}-\frac{1}{2}\left(d-\frac{2\;tz}{\sin57.5{}^{\circ}}\right)\left(\frac{d\pi }{2}-\frac{2tz}{\sin32.5{}^{\circ}}\right)}{\frac{1}{2}d\cdot \frac{d\pi }{2}}=1-f_{ZW}\;$$

Therefore, the yield stress for the polycrystalline aggregate will equal

(12) $$\sigma_{s}=\sigma_{ZW}+4.77\cdot tz\left(\sigma_{ZZ}-\sigma_{ZW}\right)d^{-1}-5.68\cdot tz^{2}\left(\sigma_{ZZ}-\sigma_{ZW}\right)d^{-2}$$

Similar to the case of a regular hexagon, the following final relation was obtained:

(13) $$\sigma_{s}=\sigma_{ZW}+9.57\cdot k\left(\sigma_{ZZ}-\sigma_{ZW}\right)d^{n-1}-22.72\cdot k^{2}\left(\sigma_{ZZ}-\sigma_{ZW}\right)d^{2n-2\;}$$

Similar calculations were made for the composite model with the grain shape of a symmetric trapezium. In this case, it was assumed that the height of a symmetric trapezium h is equal to the grain diameter d and the lower base is twice as long as the upper base. The angels in the assumed trapezium equal 75° and 105°. For such a model, the area fractions of the particular parts of the grain were calculated:

(14) $$f_{ZW}=\frac{\frac{1}{2}\left(\frac{d\pi }{2}-2.56tz\right)\left(d-2tz\right)}{\frac{1}{4}d\pi \cdot d}$$

(15) $$\;f_{ZZ}=\frac{\frac{1}{4}d\pi \cdot d-\frac{1}{2}\left(\frac{d\pi }{2}-2.56tz\right)\left(d-2tz\right)}{\frac{1}{4}d\pi \cdot d}=1-f_{ZW}\;$$

The yield stress equals

(16) $$\sigma_{s}=\sigma_{ZW}+7.12\cdot tz\left(\sigma_{ZZ}-\sigma_{ZW}\right)d^{-1}-10.24\cdot tz^{2}\left(\sigma_{ZZ}-\sigma_{ZW}\right)d^{-2}$$

Ultimately, the following relation for the yield stress of the polycrystalline aggregate was obtained:

(17) $$\sigma_{s}=\sigma_{ZW}+14.24\cdot k\left(\sigma_{ZZ}-\sigma_{ZW}\right)d^{n-1}-40.96\cdot k^{2}\left(\sigma_{ZZ}-\sigma_{ZW}\right)d^{2n-2\;}$$

## 3. Geometrical Size Effect

The other analyzed size effect is the geometrical effect. In order to consider this effect, a surface material model was applied. The aim was to determine the influence of the geometrical size effect on the changes in the flow stresses. The surface model divides the analyzed samples into two parts: the surface layer and the inner section. The yield stress can be calculated from the formula

(18) $$\sigma =\eta \sigma_{wewn}+\left(1-\eta \right)\sigma_{pow}$$

where: σ —the material’s flow stresses (MPa), η —the geometric scale coefficient (–), and $\sigma_{wewn},\;\sigma_{pow}\;$—the flow stresses for the inner and the surface grains (MPa).

**Figure 2:**
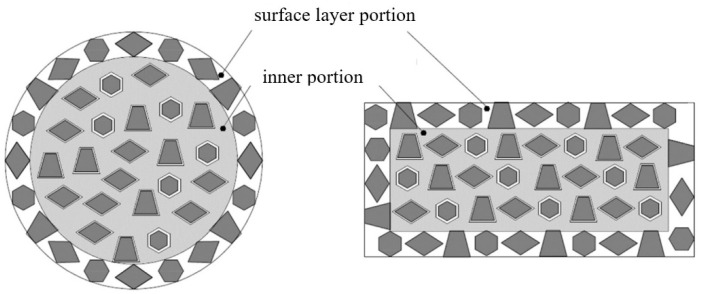
Surface layer and inner portions in a specimen section.

The grains in the inner portion of the specimen for the surface model consist of the interior and grain boundary. Therefore, the flow stresses of the inner grains are equal to the flow stress of the polycrystalline aggregate: $\sigma_{wewn}\;\;$= $\;\sigma_{s}.$ The grains located at the free surfaces have less hardening effect than the inner grains. It is assumed that in the surface layer, the phenomenon of external grain hardening occurs, so the flow stress of the surface layer in the surface model will be equal to the flow stress of the interior grain of the composite model: $\sigma_{pow}\;\;$= $\;\sigma_{ZW}$. It was assumed that the thickness of the surface layer is equal to the thickness of one grain [10]. Figure 2 shows a cross-section of a cylindrical sample with its marked layers.

The surface model will consist of different grain shapes, so the flow stresses for this model will be represented by the following formula:

(19) $$\sigma_{S}=\overset{n}{\underset{i=1}{\sum }}r\cdot \sigma_{si}$$

where: *r*—the particular shares of the grains in the structure, and $\sigma_{s_{i}}\;$—the given yield stress for the particular grain shape.

By analyzing the microsection composition of titanium Grade 2, it is possible to determine the shares of the particular grain shapes in the material. It was assumed that the rhomb-shaped grains occupy approximately 50%, the trapezium-shaped grains 40%, and the hexagon-shaped grains 10%. Presented below is an equation which takes into account the particular shares.

(20) $$\begin{array}{c} \sigma =\sigma_{ZW}+\left(0,1\cdot \frac{16}{\sqrt{3}}+0.4\cdot 14.24+0.5\cdot 9.57\right)k\eta \left(\sigma_{ZZ}-\sigma_{ZW}\right)d^{n-1}-(0.1\\ \;\;\;\;\;\;\;\;\;\;\;\;\;\;\;\;\;\;\;\;\;\cdot \frac{64}{3}+0.4\cdot 40.96+0.5\cdot 22.72)k^{2}\eta \left(\sigma_{ZZ}-\sigma_{ZW}\right)d^{2n-2\;} \end{array}$$

In order to lower the parameter *η* for the cylindrical sample, we use the geometric relation

(21) $$\eta =\frac{\frac{{\left(D-2d\right)}^{2}}{4}\cdot \pi }{\frac{D^{2}}{4}\pi }$$

Therefore, the elaborated model (21) takes into account the effects connected with the grain size and the geometrical dimensions of the sample.

## 4. Determination of the Parameters for a Model Considering Two Size Effects

In the analyzed material model (21), there are two unknowns, which are the flow stresses of the interior grain $\sigma_{ZW}$ and the boundary grain $\sigma_{ZZ}$. We assume that the flow stresses can be presented in the form of the following linear function [10]:

(22) σ=A+Bη

where:

(23) $$\begin{array}{ll} A=\sigma_{ZW};\;\;\;\;B= & \left(0.1\times \frac{16}{\sqrt{3}}+0.4\times 14.24+0.5\times 9.57\right)k\left(\sigma_{ZZ}-\sigma_{ZW}\right)d^{n-1}\\ & -(0.1\times \frac{64}{3}+0.4\times 40.96+0.5\times 22.72)k^{2}\left(\sigma_{ZZ}-\sigma_{ZW}\right)d^{2n-2\;} \end{array}$$

When there are more than two experimentally determined reinforcement curves with different geometrical scale factors η, it is possible to determine the model parameters (*A* and *B*). In order to determine the form of the linear function (22), parameters *A* and *B* from different reinforcement curves were matched in such a way so as to obtain the final form of Equation (22). In this way, we can determine the values for $\sigma_{ZW\;}\mathrm{and}\;\sigma_{ZZ}$.

## 5. Experimental Tests

In order to verify the correctness of the assumed model, an experiential compression test was performed on cylindrical samples made of titanium Grade 2 with the mean grain size of *d* = 37 µm. To guarantee each specimen has a similar deformation, a scaling factor *λ* is introduced to scale-down the specimen geometry and process parameters. In the tests, three different scale factors *λ* and *η* were applied, which have been compiled with the assumed sample dimensions in Table 1.

**Figure 3:**
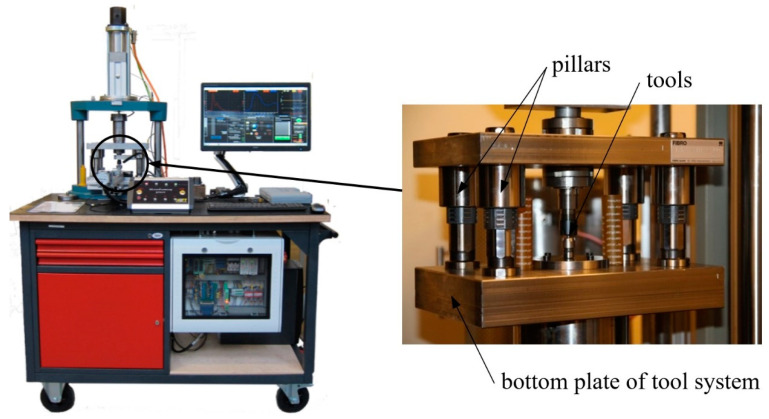
Machine for microforming processes.

The tests involved the use of a microprocessing machine constructed by the authors [18,19] (Figure 3), with a maximal force of 5000 N and a tool positioning accuracy of ±500 nanometers, equipped with an automatic feeder and with the possibility of tool work at a temperature up to 400 °C.

**Figure 4:**
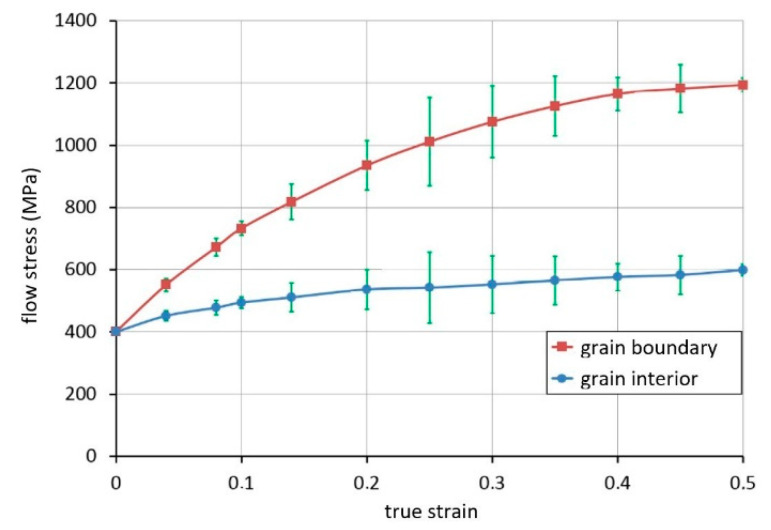
Flow curves for boundary and interior grains, a diagram obtained based on a uniaxial compression test.

With the application of the results obtained from the uniaxial compression test, the flow stresses for the interior and boundary grains were calculated based on Equation (22). According to the composite material model, the flow stress of the grain boundary is higher than that of the grain interior. The flow curves for the boundary and interior grains determined based on the calculation procedure described in Section 4 are shown in Figure 4.

## 6. FEM Simulation

FEM modeling of uniaxial compression tests was performed with the use of the MSC MarcMentat 2016 program. For the FEM calculations, the flow curves determined based on Equation (22) were applied. An elastic–plastic model with isotropic hardening was used. The model used symmetry in respect to the axis of the cylindrical sample, and rigid tools were applied. For the creation of the final element grid, the four-node element number 10 was applied and the Coulomb friction model was used (µ = 0.2) [20].

**Figure 5:**
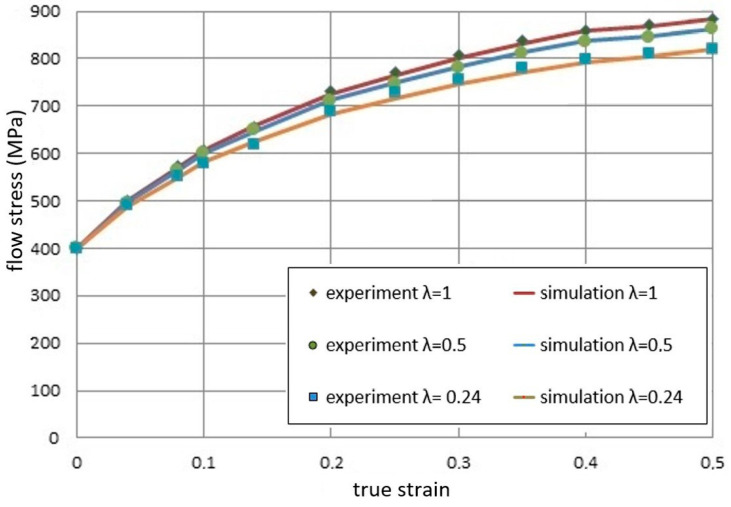
Comparison of the stress–strain curves obtained from the experiment and the simulation.

Figure 5 shows a comparison of the experimental results and the theoretical results obtained based on the constitutive model presented earlier.

In the experiment, the grain size is constant, and the dimension of the billet as well as other conditions is varied according to the similarity principle. The miniaturization resulted in lower flow stresses. A very good agreement of the experimental results and the FEM simulation results of three titanium samples made with different scale factors λ was obtained. For larger deformations, we can observe the influence of the size effect on the obtained yield stress values. The phenomenon of decreasing flow stress curves occurs. The model can be used to reveal the effect of geometry size on the deformation of bulk metals.

**Figure 6:**
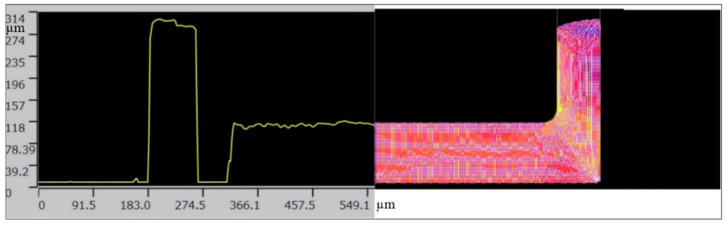
Comparison of the geometry of the backward extrusion workpiece obtained from the experiment and the simulation.

The material model was also used for a simulation of the backward microextrusion process of titanium Grade 2. The analysis covered the geometrical shape of the product obtained from the simulations with the use of a material model taking into account the size effect and from the experiment performed on the microforming machine (Figure 6). The experimental measurements were conducted with the use of a confocal microscope Olympus LEXT 3D Measurine Laser Microscope OLS 4000.

**Figure 7:**
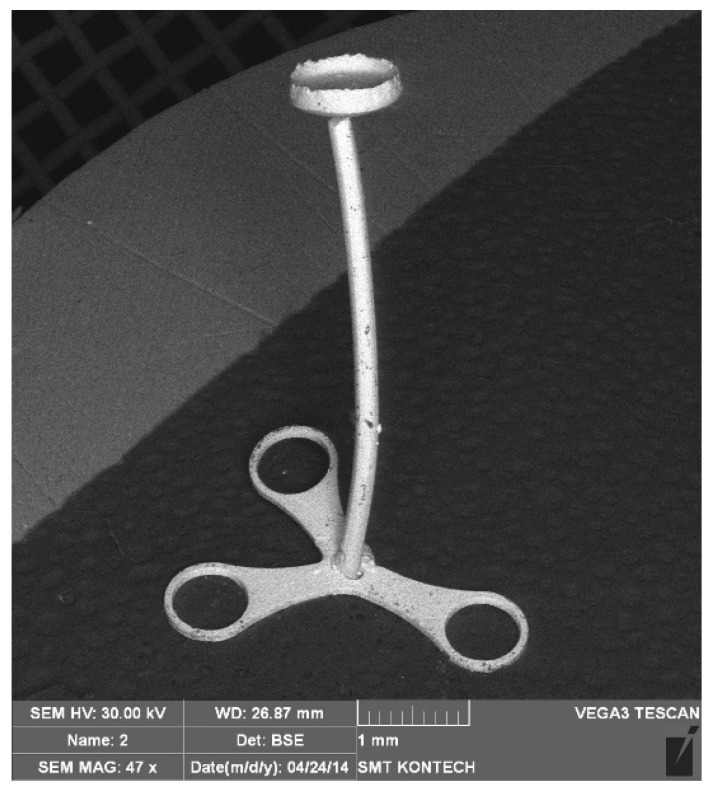
Experimentally made ossicle implant.

We can see a good representation of the shape of the workpiece obtained in the FEM model (Figure 6). The numerical simulations of the microextrusion and micropressing processes were helpful in the development of tools for microforming a TOPR-type implant, which is shown in Figure 7.

The elaborated material model considering the size effects ensured the proper FEM modeling of the microformed components of an ossicle implant.

## 7. Conclusions

The size effect is considered as an important factor in microforming processes. This study applied a constitutive model taking into account two size effects: one is attributed to the grain size and the other is related to the geometrical dimensions of the sample. The model consists of a surface model and a composite material model. Additionally, the surface model considered different grain shapes present in the actual polycrystalline material such as pure titanium Grade 2. Titanium is often used for the manufacture of microparts in biomedical devices and implants.

The novelty of the work is the use of a few selected grain shapes: a regular hexagon, a rhomb, and a symmetric trapezium. The model parameters (A and B) determined for samples made of titanium Grade 2 were used to describe the hardening curves applied in the FEM simulations performed for the process of uniaxial compression. The results obtained from the experiments and the simulation are similar.

The developed material model can be used for the description of a wide range of materials with diversified grain shapes and should be applied during the FEM modeling of microforming processes in order to consider the various size effects occurring during deformation. Examples of such materials are copper and aluminum alloys. They contain selected grain shapes used in the new material model. The developed material model was used with success for a simulation of the backward microextrusion process component of an ossicle implant.

## Figures and Tables

**Table 1:** Geometrical dimensions and process parameters for micro-upsetting.

Sample Data/Number	1	2	3
Height (mm)	3.00	1.50	0.72
Diameter (mm)	2.00	1.00	0.48
Compression rate (mm/s)	0.007	0.0035	0.0016
Scale factor (*λ*)	1	0.5	0.24
Geometrical scale factor (*η*)	0.93	0.86	0.73

## A List of Acronyms

Grade 2	type of titanium according to ASTM F67 (ISO 5832-2), material for medical applications.
FEM	finite element method.
TOPR	total ossicular replacement prosthesis.
